# Insights into atypical segmental layer thicknesses and phase retardation in thick corneas using ultrahigh-resolution polarization-sensitive optical coherence tomography

**DOI:** 10.1186/s40662-024-00391-4

**Published:** 2024-07-15

**Authors:** Rahul P. Patil, Rohit Shetty, Pooja Khamar, Yash G. Patel, Raghav R. Narasimhan, Anushree A. Bhatkal, Christopher K. Hitzenberger, Michael Pircher, Rudy M. M. R. Nuijts, Abhijit Sinha Roy

**Affiliations:** 1 Imaging, Biomechanics and Mathematical Modelling Solutions Lab, Narayana Nethralaya Foundation, Narayana Nethralaya, Bangalore, India; 2 Department of Cornea and Refractive Surgery, Narayana Nethralaya, Bangalore, India; 3 Center for Medical Physics and Biomedical Engineering, Medical University of Vienna, Vienna, Austria; 4 University Eye Clinic Maastricht, Maastricht University Medical Center, Maastricht, The Netherlands

**Keywords:** Thick cornea, Keratoconus, Collagen, Phase retardation, Stroma, PS-OCT, Birefringence

## Abstract

**Background:**

Accurately assessing corneal structural status is challenging when thickness deviates from the average. Polarization-sensitive optical coherence tomography (PS-OCT) measures tissue-specific polarization changes, providing additional contrast for accurate segmentations and aids in phase retardation (PR) measurements. Previous studies have shown PR's effectiveness in identifying sub-clinical keratoconus (KC) in asymmetric cases. Thus, this study aims to assess PR distribution in thick corneas with and without KC.

**Methods:**

In this retrospective and cross-sectional study, 45 thick corneas from 30 Asian-Indian subjects, categorized into healthy (n = 26) and KC (n = 19) groups were analyzed. All eyes underwent standard clinical evaluations, tomographic assessments, and corneal biomechanics measurements. PR and individual layer thicknesses were measured using custom-designed ultrahigh-resolution PS-OCT. PR en-face maps were generated. Individual layer thicknesses and PR analysis was conducted across multiple zones, extending up to 8–10 mm in diameter. All eyes in the study had not undergone interventions, received topical medications, or had previous corneal disease history.

**Results:**

Significant differences were found in spherical and cylindrical powers, keratometry, pachymetry, and biomechanical indices (all *P* < 0.01). Thickness profiles from PS-OCT showed significant differences in the 4–8 mm zones only. Bowman's layer thickness significantly differed only in the central 2 mm zone (*P* = 0.02). The median PR values showed marginal differences in the central 2 mm zone (*P* = 0.0565). Additionally, there were significant differences observed in the 2–4 mm and 4–6 mm zones (*P* = 0.0274 and *P* = 0.0456, respectively). KC eyes exhibited an atypical PR distribution and corneal thinning, while normal eyes maintained a uniform Bowman’s layer thickness and PR maps with larger areas of higher PR.

**Conclusion:**

The study revealed distinctive PR distribution in thick corneas among healthy and KC groups. Using an ultrahigh-resolution PS-OCT the significance of Bowman's layer thickness in these groups was also emphasized. The study offered potential improvements in clinical diagnostics by enhancing our understanding of corneal structure and its altered function.

**Supplementary Information:**

The online version contains supplementary material available at 10.1186/s40662-024-00391-4.

## Background

Diagnosing and classifying various corneal diseases involve adjunct criteria like topography, tomography, and biomechanical properties [1–3]. However, deviations from normal thickness (~ 530 µm) make precise diagnosis challenging [4–7]. A weak but positive correlation between corneal thickness and curvature in healthy corneas has been reported [4]. Such an association may contribute to inconclusive results on corneal topography. Additionally, any underlying structural changes can impact the biomechanical properties of the cornea [5]. Naturally thin (< 500 µm) or thick corneas (> 550 µm) can also lead to misdiagnosis of glaucoma, since intraocular pressure measurements (IOP) can be influenced by central corneal thickness (CCT) [6, 7]. Corneal thickness is crucial in refractive surgeries, with thick corneas allowing for greater corrections. Thin corneas pose challenges and may increase the risk of postoperative ectasia and other complications. Artificial intelligence (AI)-driven biomechanical simulations that determine the risk of ectasia revealed post-surgery ectasia in eyes with CCT > 530 µm, challenging the notion that ectasia is exclusive to thin corneas [8]. Therefore, there is a need for approaches like ultrahigh-resolution PS-OCT, which surpass conventional diagnostic methods that assess only the shape and thickness [9].

PS-OCT provides additional contrast by recording of tissue specific polarization changes [10]. Thereby, enabling measurement of phase retardation (PR) caused by form birefringence in aligned and reinforcing collagen fibrils [9–12]. We have evaluated asymmetric keratoconus in an earlier study and the findings were clearly able to differentiate between sub-clinical and clinical disease based on PR mapping [9]. Repeatability of corneal PR maps across healthy volunteers was also demonstrated [12]. In a recent study, posterior scleral birefringence was evaluated as a biomarker for predicting the risk of myopia progression [13]. By comparing PS-OCT with transmission electron microscopy (TEM), higher birefringence in PS-OCT images corresponded to more aligned fibers in the posterior sclera [13]. Additionally, increased birefringence was associated with larger average collagen fiber diameter, indicating higher collagen content and thicker lamellae [13]. These studies demonstrate and establish the clinical importance and efficacy of PS-OCT as a powerful tool for non-invasive evaluation of corneal microstructure. Building upon our previous study on thin corneas, this study focuses on analyses of PR mapping, specifically in thick corneas with and without the presence of KC.

## Methods

This retrospective, observational and cross-sectional study included a total of 45 eyes belonging to 30 Asian-Indian subjects. The study was approved by the Narayana Nethralaya Ethics Committee (ECR/187/INST/KAR/2013), Bangalore, India, and followed the tenets of the Declaration of Helsinki. All eyes in this study were subjected to routine clinical assessment (slit lamp), measurement of tomography using Pentacam® (Oculus Optikgeräte GmbH, Germany), MS-39 (Costruzione Strumenti Oftalmici, Italy) and biomechanical assessment using Corneal Visualization Scheimpflug Technology (Corvis® ST, Oculus Optikgeräte, GmbH, Wetzlar, Germany). For further analysis, eyes were classified into two groups: healthy and KC. The healthy thick cornea (> 550 µm) group consisted of 26 healthy eyes with normal quantitative parameters and topography patterns observed during Pentacam® and slit lamp examinations. In addition, we included 19 eyes in the KC group which were grades 1 and 2 on the Amsler-Krumeich classification. When comparing the KC group with healthy thick corneas, even though the CCT in the KC group did not exceed 550 µm in any eye, the pachymetry indicated overall thick corneas as the KC corneas group had a minimum thickness greater 500 µm. This suggested that the lower thicknesses (under 550 µm) were due to focal thinning characteristic of KC. The diagnosis of KC was established using slit-lamp observations, asymmetric tomographical features and steepening of the anterior and posterior corneal surfaces. All eyes included in the study had not undergone any interventions or received topical medications before the analyses. Participants with conditions such as pregnancy, previous corneal disease, thyroid gland dysfunction, other corneal co-pathologies, atopy, atopic dermatitis, trisomy were excluded from the study.

PS-OCT imaging was performed by a single operator using our custom-built ultrahigh-resolution OCT scanner that operated at a center wavelength of 840 nm and 100 nm spectral bandwidth [9, 14]. A scan field of 10 mm diameter was achieved using conical scanning that provided a near uniform signal strength throughout the cornea [11]. The total incident power on the cornea was set to 1 mW. All scans were acquired at 30 kHz A-scan rate using a raster (rectangular) scan pattern of 64 B-scans with 1024 A-scans per B-scan in less than 3 s. The polarization state of light in sample and reference arms was set using quarter-wave plates at 45 and 22.5 degrees, respectively. This allowed circularly polarized light to illuminate the sample and provided equal reference power in the two orthogonal polarization channels. The polarization-sensitive information was derived in the form of reflectivity (R) and PR using the following equations:

1 $$\text{R}\left(\text{z}\right) \sim {\text{A}}_{1}{(\text{z})}^{2}+{\text{A}}_{2}{(\text{z})}^{2}$$

2 $$\text{PR}\left(\text{z}\right)=\text{arctan}\left(\frac{{\text{A}}_{2}(\text{z})}{{\text{A}}_{1}(\text{z})}\right)$$

where ${\text{A}}_{1}$ and ${\text{A}}_{2}$ represented the amplitudes of the complex signals obtained using Fourier transform of the spectral data of the two orthogonal polarization channels and z was the depth coordinate [15]. Additional details about our custom-built PS-OCT can be found in our previous works [9, 14].

From PS-OCT, PR *en-face* maps were generated from the posterior surface as this layer is polarization preserving and enables the observation of polarization changes introduced by the arrangement of collagen lamellae in the stroma [10, 11]. Corneal layer thickness profiles across five different zones between 2–10 mm were also generated. Further details on generation of layer thickness mapping can be found in our previous work [12]. The PR and thickness *en-face* maps were smoothened by applying a floating average filter equivalent to 1 mm × 1 mm area of the cornea. The 2D color-filled contour plots were generated for the smoothened PR *en-face* maps. The Bowman's layer is known to be clearly visible up to approximately 9 mm before thinning and merging into the cornea. To ensure segmentation uniformity for epithelium and Bowman’s layer thickness mapping, we considered an 8 mm field along the x- and y-axes. Meanwhile, PR, stroma and total corneal thickness data were extended to a field size of 10 mm (horizontal) × 8 mm (vertical). Further, the color scales were fixed according to the global consensus of corneal thickness ranges and were are not varied according to the population. From MS-39, the following indices were analyzed: flat keratometry (K1), steep keratometry (K2), maximum keratometry (Kmax). While from Pentacam® and Corvis ST®, the following indices were analyzed: Corvis biomechanical index (CBI), tomographic and biomechanical index (TBI), stiffness parameter A1 (SPA1), stress–strain index (SSI), horizontal white to white (HWTW), Belin/Ambrósio Deviation (BAD-D) and biomechanical corrected IOP (bIOP). All OCT images were analyzed using custom built software. All parameters were summarized as median with 95% confidence interval after assessment of normality of distribution (Kolmogorov–Smirnov test). Differences in parameters across groups were assessed using the Mann–Whitney test. A *P* value less than 0.05 was considered statistically significant. MedCalc v.22.005 (MedCalc Inc., Ostend, Belgium) software was used for statistical analyses.

## Results

Cohort demographics for thick corneas included in the study are summarized as median and its corresponding 95% confidence interval (Table 1). Median age between the two groups was significantly different (*P* = 0.03) but this was clinically insignificant. The spherical and cylindrical powers were significantly different between the groups (*P* < 0.01). Keratometry, pachymetry and biomechanical indices were significantly different between the groups (*P* < 0.01). However, no significant difference (*P* > 0.05) was observed in biomechanically corrected IOP (bIOP), horizontal white-to-white (HWTW) and stress–strain index (SSI). Table 2 shows corneal sub-layer thickness profiles estimated from PS-OCT at various annulus zones (2 mm, 2–4 mm, 4–6 mm, 6–8 mm, and 8–10 mm) between healthy thick corneas and corneas with KC. In the central 2 mm zone, the median epithelial thickness for healthy thick corneas was 52.49 µm (range, 50.36 to 54.22 µm), while for KC corneas it was 50.62 µm (range, 44.74 to 52.66 µm).

**Table 1:** Demographics for healthy thick (*n* = 26) and KC thick eyes (*n* = 19). Values represented as median with 95% confidence interval

Parameters	Healthy thick corneas	KC thick corneas	*P* value*
Age (years)	28 [25.65, 30.00]	25 [22.00, 28.00]	0.03
Sphere (D)	− 2.25 [− 2.50, − 2.00]	0.00 [− 1.15, 0.50]	< 0.01
Cylinder (D)	− 0.50 [− 0.61, 0.00]	− 3.00 [− 3.25, − 1.75]	< 0.01
UDVA (logMAR)	0.00 [0.00, 0.74]	0.54 [0.36, 0.86]	< 0.01
CDVA (logMAR)	0.00 [0.00, 0.00]	0.18 [0.05, 0.18]	< 0.01
Curvature parameters			
K1 (D)	42.62 [42.07, 43.58]	44.94 [43.91, 47.18]	< 0.01
K2 (D)	43.85 [43.42, 44.57]	51.11 [49.68, 54.10]	< 0.01
Kmax sagittal (D)	44.05 [43.74, 44.64]	51.40 [50.26, 54.42]	< 0.01
Biomechanical parameters			
CBI	0.20 [0.10, 0.24]	0.92 [0.49, 0.97]	< 0.01
TBI	0.28 [0.024, 0.40]	1 [1.00, 1.00]	< 0.01
SPA1	119.51 [114.87, 129.69]	93.03 [86.05, 101.67]	< 0.01
SSI	0.94 [0.87, 1.028]	0.93 [0.71, 1.09]	0.66
HWTW (mm)	12 [11.84, 12.10]	12 [11.90, 12.22]	0.78
BAD-D	1.06 [0.54, 1.47]	6.39 [5.07, 7.86]	< 0.01
bIOP (mmHg)	15.15 [14.15, 16.25]	15.5 [14.38, 15.90]	0.82

*UDVA* = uncorrected visual acuity; *CDVA* = corrected distance visual acuity; *K1* = flat keratometry; *K2* = steep keratometry; *Kmax* = maximum keratometry; *CBI* = Corvis biomechanical index; *TBI* = tomographic biomechanical index; *SPA1* = stiffness parameter A1; *SSI* = stress–strain index; *HWTW* = horizontal white to white; *BAD-D* = Belin/Ambrósio enhanced ectasia display deviation; *bIOP* = biomechanically corrected intraocular pressure

^*^Mann–Whitney test

**Table 2:** Epithelial layer, Bowman’s layer, stroma, and total corneal thickness measurements between healthy (n = 26) and keratoconus (KC) eyes (n = 19)

Zone	Healthy thick corneas	KC thick corneas	*P* value*
Epithelial layer thickness (µm)			
2 mm	52.49 [50.36, 54.22]	50.62 [44.74, 52.66]	0.08
2 to 4 mm	53.11 [50.94, 54.64]	52.42 [50.76, 55.50]	0.89
4 to 6 mm	52.98 [50.88, 53.69]	53.76 [52.86, 56.86]	0.04
6 to 8 mm	51.37 [49.37, 53.64]	53.83 [51.97, 56.25]	0.02
Bowman’s layer thickness (µm)			
2 mm	16.41 [15.97, 16.97]	15.47 [14.91, 16.47]	0.02
2 to 4 mm	16.53 [16.09, 17.00]	15.67 [15.14, 16.32]	0.06
4 to 6 mm	16.31 [16.08, 16.68]	16.22 [15.23, 16.61]	0.51
6 to 8 mm	16.08 [15.75, 16.32]	16.31 [15.33, 16.60]	0.49
Stroma thickness (µm)			
2 mm	476.31 [474.24, 486.22]	449.78 [422.75, 458.47]	0.01
2 to 4 mm	497.26 [493.63, 502.64]	475.4 [443.90, 481.88]	0.02
4 to 6 mm	538.64 [529.71, 551.20]	511.61 [486.22, 529.90]	0.01
6 to 8 mm	584.4 [571.05, 599.91]	549.1 [528.22, 571.82]	0.01
8 to 10 mm	620.48 [597.75, 635.78]	587.87 [560.45, 602.09]	0.01
Total corneal thickness (µm)			
2 mm	548.78 [545.99, 554.51]	521.92 [488.88, 527.15]	< 0.001
2 to 4 mm	570.13 [564.91, 574.38]	547.2 [508.82, 554.16]	< 0.001
4 to 6 mm	610.63 [602.21, 620.17]	587.32 [554.88, 601.80]	0.01
6 to 8 mm	655.15 [639.64, 668.96]	624.83 [598.27, 644.25]	0.01
8 to 10 mm	688.79 [667.00, 703.56]	658.57 [628.23, 676.49]	0.01

^*^Mann–Whitney test

Median values are depicted with 95% confidence intervals. Epithelial layer and Bowman’s layer maps cover a field size of 8 × 8 mm, while stroma and total corneal thickness maps extend across a 10 × 8 mm field

Surprisingly, the difference between the two groups was marginally insignificant (*P* = 0.08). In the 2 to 4 mm zone, the median thickness of the epithelial layer was 53.11 µm (range, 50.94 to 54.64 µm) for healthy thick corneas and 52.42 µm (range, 50.76 to 55.5 µm) for KC corneas (*P* = 0.89). However, significant differences between healthy thick corneas and KC corneas in the 4 to 6 mm (*P* = 0.04) and 6 to 8 mm zones (*P* = 0.02) were observed. The median Bowman's layer thickness of the central 2 mm zone was 16.41 µm (range, 15.97 to 16.97 µm) for healthy thick corneas and 15.47 µm (range, 14.91 to 16.47 µm) for KC thick corneas (*P* = 0.02). The remaining zones had similar thicknesses between the two groups (*P* > 0.05). For the stroma and corneal thickness, significant (*P* < 0.05) differences between the two groups were observed across all five zones. Overall, analyses showed significant differences (*P* < 0.05) in the thickness of the peripheral zones (epithelial and stroma) as well as total corneal thickness between healthy thick corneas and KC corneas. However, only the central zones in the Bowman’s layer were significantly different between the two groups (*P* < 0.05).

**Fig. 1:**
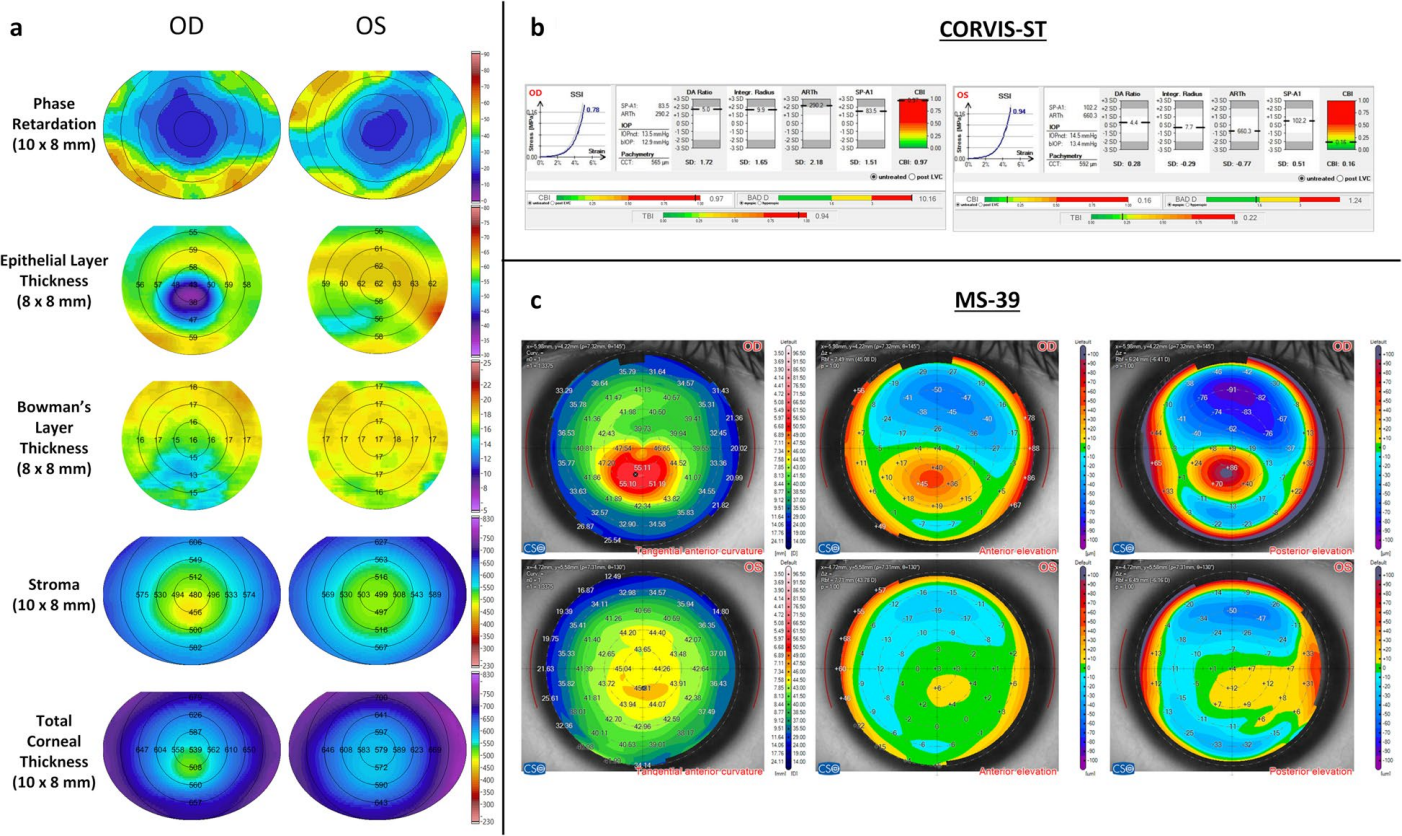
Representative case featuring an asymmetric KC thick cornea. **a** Maps of phase retardation (PR) (10 × 8 mm), epithelium layer (8 × 8 mm), Bowman's layer (8 × 8 mm), stroma (10 × 8 mm), and total corneal thickness (10 × 8 mm), acquired using our custom-built ultrahigh resolution polarization-sensitive optical coherence tomography (PS-OCT). **b** Biomechanical indices from Corvis-ST. **c** Maps of tangential anterior curvature, anterior elevation, and posterior elevation obtained from a hybrid tomographer (MS-39). All maps derived from PS-OCT (**a**) and MS-39 (**c**) are overlaid with radial circles at 2 mm intervals

Table 3 shows the median PR values with 95% confidence interval from different regions of the cornea. In the central 2 mm zone, the median PR for healthy thick and thick KC corneas was 25 degrees (range, 23.35 to 30.29 degrees) and 23 degrees (range, 22.57 to 25.02 degrees), respectively (*P* = 0.0565). In the 2 to 4 mm zone, the median PR for healthy thick corneas and thick KC cornea was 30 degrees (range, 27.44 to 33.45 degrees) and 28 degrees (range, 25.86 to 28.07 degrees), respectively (*P* = 0.0274). A similar trend of lower PR in KC was observed in the 4 to 6 mm zone (*P* = 0.0456) as well as in the 6 to 8 mm and 8 to 10 mm zones (*P* > 0.05). While the median data showed some differences between the two groups, the challenge in evaluating these KC thick corneas is illustrated in Fig. 1.

**Table 3:** Phase retardation (degrees) measurements conducted from the central cornea to the periphery in both healthy (n = 26) and keratoconus (KC) eyes (n = 19)

Zones	Healthy thick corneas	KC thick corneas	*P* value*
2 mm	24.92 [23.35, 30.29]	23.21 [22.57, 25.02]	0.0565
2 to 4 mm	30.19 [27.44, 33.45]	27.46 [25.86, 28.07]	0.0274
4 to 6 mm	39.03 [36.32, 41.51]	36.34 [34.58, 38.63]	0.0456
6 to 8 mm	46.02 [43.85, 47.13]	43.81 [41.21, 45.12]	0.0848
8 to 10 mm	50.84 [49.10, 52.72]	47.49 [44.64, 49.40]	0.0980

^*^Mann–Whitney test

Median values are illustrated with 95% confidence intervals. Phase retardation maps cover a field size of 10 × 8 mm

Figure 1 shows the measurements of the PS-OCT, Corvis® ST and MS-39 of a patient with one eye having clinical KC (OD) and the other eye (OS) having normal topography (Fig. 1c). The OD eye was included in the KC thick cornea group. The PS-OCT data clearly shows an abnormal PR distribution relative to a normal healthy cornea (Fig. 1a) [9, 12]. Further, there is marked thinning in the epithelium and Bowman’s layer of the OD eye. The OS eye also exhibited epithelial thinning in the inferior zone as depicted in Fig. 1a but maintained a normal Bowman’s layer thickness. Figure 1b shows the Corvis® ST measurements for both the OD and OS eyes. While the CBI, TBI and SSI of the OD eye indicated disease, the OS eye did not show any signs of disease despite the abnormal PR and epithelium thinning indicated on PS-OCT (Fig. 1a). Figure 1c presents the corresponding MS-39 maps for OD (top row) and OS (bottom row). The OD eye showed evident KC characteristics such as high anterior curvature (Kmax: 56.69 D) and elevated measurements in both anterior and posterior surfaces. In contrast, the fellow eye (OS) exhibited a fairly uniform curvature (Kmax: 45.31 D) and modest elevations on the anterior and posterior surfaces, indicating healthy status.

## Discussion

A thick or thin cornea alone does not necessarily mean that the cornea is unhealthy as the cornea can be naturally thick or thin as a result of genetics or other factors. However, a thick or thin cornea may be a sign of an underlying condition or may indicate a higher risk for certain eye conditions such as KC [9, 16]. Besides such conditions, it can also be challenging to treat and diagnose thin and thick corneas especially while evaluating IOP measurements or when presented for refractive procedures [7, 17]. Another confounder is the lack of an universally accepted standard definition for thin, average and thick corneas [18, 19]. Further, different modalities deliver varying thickness measurement values for the same cornea with an approximate variance observed to be ± 20 µm [20–22]. Similarly, a recent study centered on the ethnic variations of CBI which is a key biomechanical index, reported a wide range of cut-off for CBI (0.27 to 0.76) for detection of KC as against the original cut-off of 0.5 [23]. In Fig. 1b, OD and OS had a CBI of 0.97 and 0.16, respectively. Thus, CBI was not abnormal in the OS eye despite epithelium thinning and an abnormal PR map (Fig. 1a). Interestingly, biomechanical indices (bIOP and SSI), which are known to be independent of CCT, were not significantly different between the two eyes [24, 25]. In this study, the 95% confidence interval for CBI and TBI was as high as 0.24 and 0.4, respectively, for healthy thick corneas. Hence, the clinical evaluation of PR maps could be useful to derive important information pertaining to the alignment of corneal collagen fibers.

As a population, Table 1 shows outcomes between the healthy and KC thick corneas agreeing to group definitions. To the best of our knowledge, no previously published data has investigated sample demographic variations in refractive error, keratometry, and biomechanical indices, and their implications on PR mapping. However, the segmental layer thicknesses (Table 2) and median PR (Table 3) show some dissimilarity between the healthy and KC thick corneas similar to the patient described in Fig. 1. Therefore, a combined evaluation of corneal tomography, PR and corneal biomechanical parameters may provide an improved evaluation of thick corneas. The HWTW provides a geometric assessment of the cornea but is limited by its assessment only along one meridian [22]. We recently showed that a small HWTW and corneal diameter along other meridians can result in the misinterpretation of steep anterior corneas with normal thickness as early KC [22]. Hence, conventional tomography of a cornea may provide incomplete information about the presence or absence of disease [26].

Table 2 clearly showed that the epithelium and Bowman’s layer are locally thinner in some regions relative to the thick KC corneas. Further, the thickness of the stroma was lower globally due to disease in KC thick corneas. KC is often considered to be bilateral and asymmetric in nature [27]. Figure 1a shows the thinning of the epithelium, Bowman’s layer, and stroma in both the KC thick cornea (OD) and fellow eye (OS). Also in KC, Bowman's layer exhibits breaks, leading to compensatory epithelial thickening in the regions of Bowman’s thinning [28]. Therefore, Bowman’s layer may have a protective role in corneal wound healing, restoration of transparency and re-innervation of nerves [29]. Bowman’s layer thickness can be potentially used as a disease screening marker along with quantitative mapping of the PR to provide valuable insight beyond conventional tomography into disease progression.

**Fig. 2:**
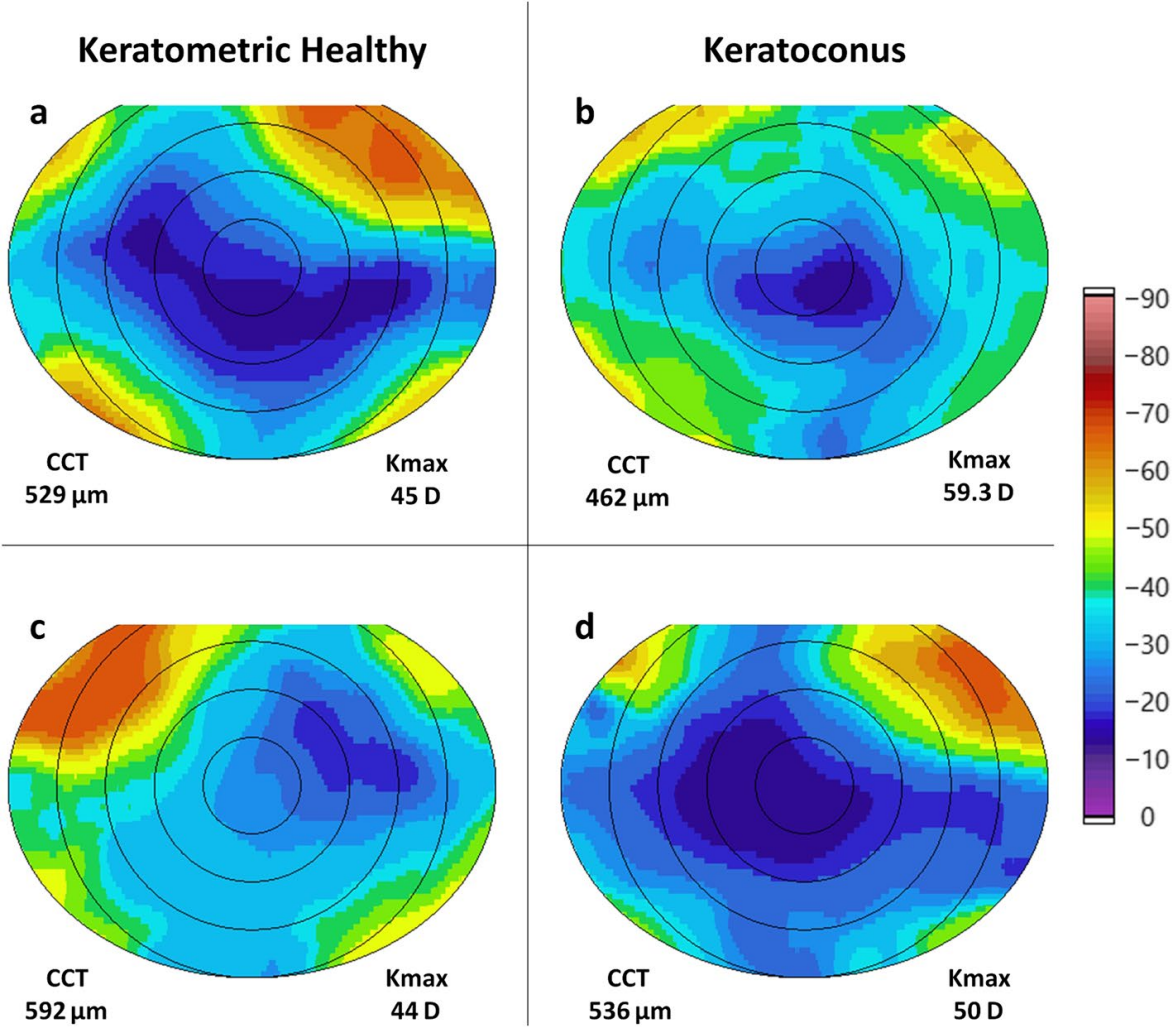
Representative phase retardation *en-face* maps of (**a**) healthy cornea with normal thickness, (**b**) keratoconus cornea, (**c**) healthy thick cornea and (**d**) thick cornea with keratoconus. “Healthy” with respect to keratometry values. Phase retardation (PR) maps are represented in a 10 × 8 mm field size. All maps are overlaid with radial circles at 2 mm intervals. CCT, central corneal thickness; Kmax, maximum keratometry

Morevoer, we compared PR maps from corneas in our previous work on thin corneas (< 500 µm) and the thick cornea cohort presented in this paper (Fig. 2) [9]. Observations were comparable with respect to contralateral eyes exhibiting a near mirror symmetry of PR distribution, as demonstrated by *ex-vivo* X-ray diffraction studies and *in-vivo* PS-OCT studies in human corneas [3, 9, 11, 12]. It was interesting to note that healthy normal thickness and healthy thin corneas appeared to have a homogenous PR distribution following the quasi-rhombus pattern with PR increasing from the center to periphery [9]. Healthy thick corneas exhibited an atypical PR distribution (Fig. 2c) rather similar to that previously seen in corneas diagnosed with frank KC (Fig. 2b), but with regions of higher PR. On the other hand, thick corneas which were tomographically indicative of KC, exhibited uniformly distributed PR (Fig. 2d) like that of healthy corneas with normal thickness (Fig. 2a). Our findings regarding the distribution of PR in thin and thick corneas suggested that changes in underlying structures may be more pronounced in thicker corneas, which naturally have a larger tissue volume. Therefore, large study cohorts representing diverse population demographics are required to further understand the essential relationship between corneal thickness and underlying structures for comprehending microstructural changes in the cornea.

Looking ahead, it would also be interesting to showcase PR mapping of various grades of keratoconus, classified according to the existing grading system. A potential acquisition-related limitation of this study could be the small sampling density of B-Scans (64 scans) due to the slow A-Scan rate of 30 kHz. It would have been more advantageous if we had a higher density of B-Scans to enhance the robustness of our findings. Another avenue to explore is the interplay between corneal thickness, stromal hydration, underlying structures, and their effects on PR. Prior research has established links between stromal thickness, retardation, refractive index, and hydration in animal corneas (specifically rabbit and cat corneas) [30–32]. While these findings provide a theoretical foundation, further prospective research documenting pachymetry, biomechanical and refractive changes over time and its correlation with PR changes is required to establish the applicability of this relationship to human corneas.

## Conclusion

This study demonstrated the PR distribution mapping in thick corneas across healthy and KC groups. Differences in PR maps were evident between healthy and KC thick corneas, specifically in the central regions up to the 6 mm diameter zone. However, these observations were atypical and remarkably different from our previous work on thin corneas. Future studies are needed to address these differences. PS-OCT has the potential to provide a deeper understanding of ocular tissues beyond traditional tomography and biomechanical indices and can be used for clinical diagnosis and treatment planning. Furthermore, additional research is required to study corneas from a more diverse population, encompassing varying thicknesses, to establish correlations between parameters like thickness, refractive index, and birefringence.

### Supplementary Information

Supplementsry Material 1.

## Data Availability

The data that support the findings of this study are available from the corresponding author upon reasonable request.

## Abbreviations

IOP: Intraocular pressure
CCT: Central corneal thickness
PS-OCT: Polarization-sensitive optical coherence tomography
PR: Phase retardation
KC: Keratoconus
TEM: Transmission electron microscopy
K1: Flat keratometry
K2: Steep keratometry
Kmax: Maximum keratometry
CBI: Corvis biomechanical index
TBI: Tomographic and biomechanical index
SPA1: Stiffness parameter A1
SSI: Stress strain index
HWTW: Horizontal white to white
BAD-D: Belin/Ambrósio enhanced ectasia display deviation
bIOP: Biomechanical corrected IOP
µm: Micro-meter
mW: Milli-Watts
kHz: Kilo-hertz
nm: Nano-meter
