# Supplementary figures and images for: Insights into atypical segmental layer thicknesses and phase retardation in thick corneas using ultrahigh-resolution polarization-sensitive optical coherence tomography
Source: Eye Vis (Lond). 2024 Jul 15;11:30. doi: 10.1186/s40662-024-00391-4 (PMC11247896; doi:10.1186/s40662-024-00391-4)

## Slide 1
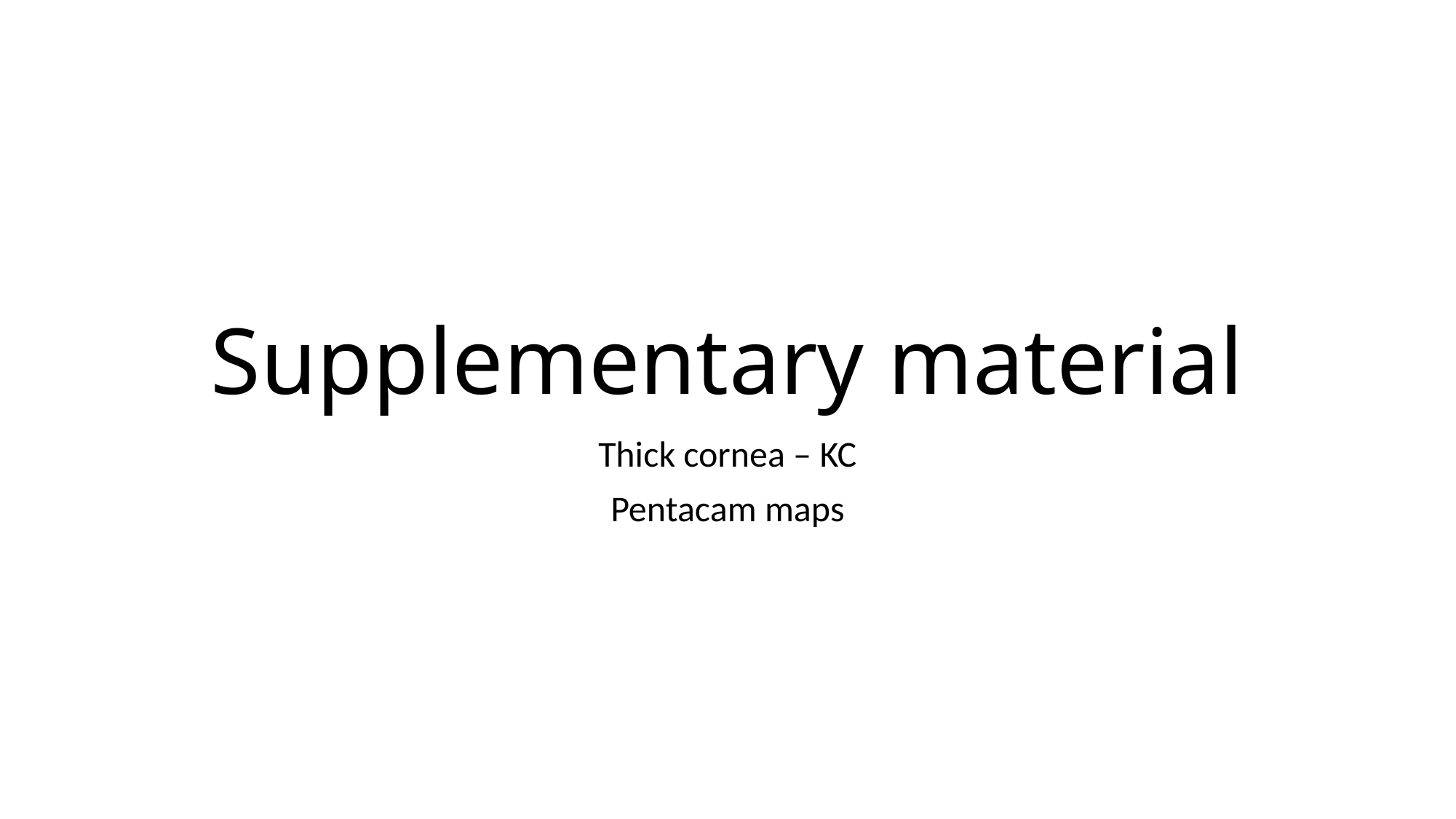

# Supplementary material
Thick cornea – KC
Pentacam maps

## Slide 2
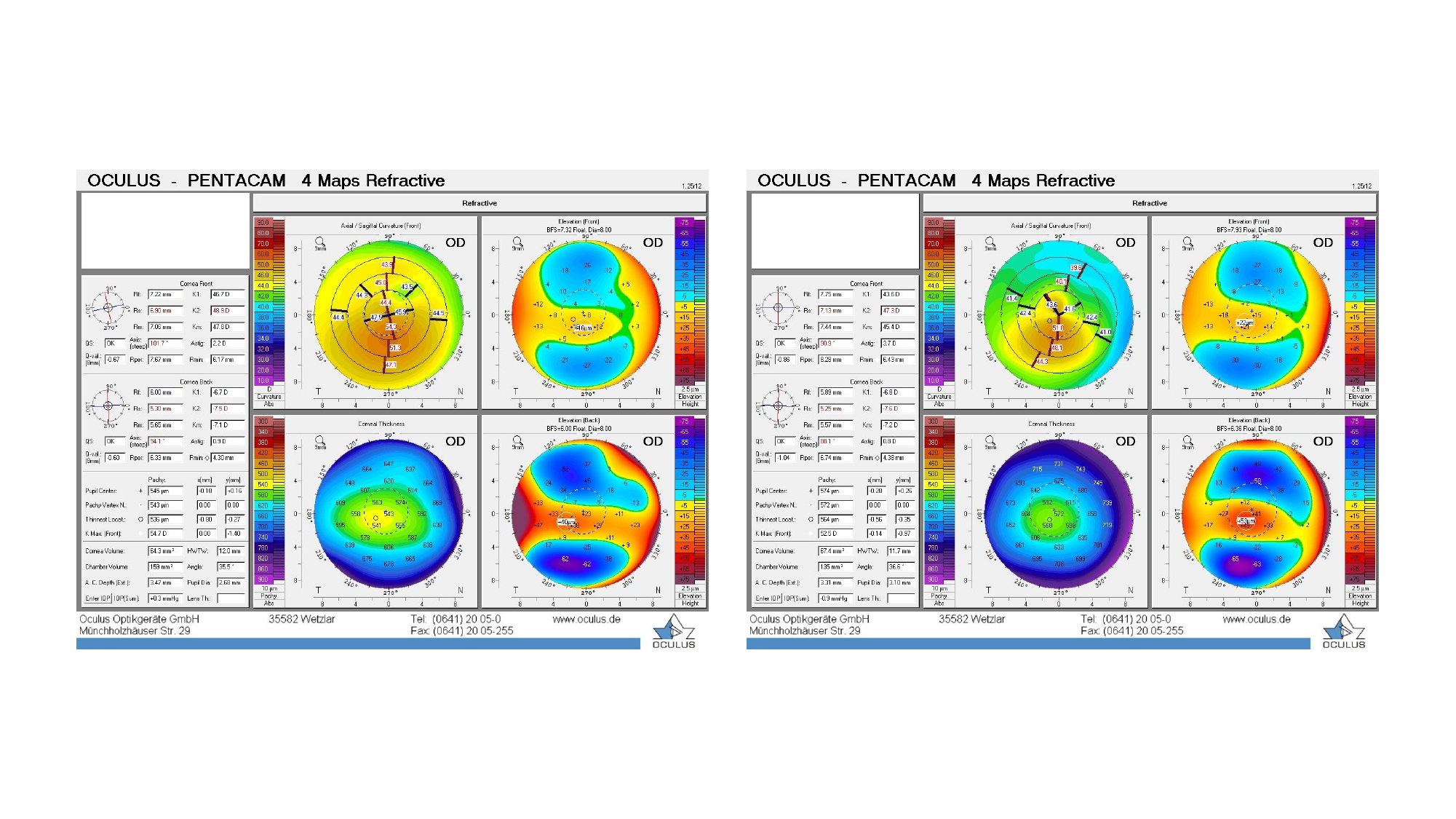

Supplement: Supplementary file 1 — Supplementsry Material 1. [file 40662_2024_391_MOESM1_ESM.pptx]
